# Cross-reactivity between vaccine antigens from the chitin deacetylase protein family improves survival in a mouse model of cryptococcosis

**DOI:** 10.3389/fimmu.2022.1015586

**Published:** 2022-09-28

**Authors:** Maureen M. Hester, Lorena V. N. Oliveira, Ruiying Wang, Zhongming Mou, Diana Lourenco, Gary R. Ostroff, Charles A. Specht, Stuart M. Levitz

**Affiliations:** ^1^ Department of Medicine, The University of Massachusetts Chan Medical School, Worcester, MA, United States; ^2^ Program in Molecular Medicine, The University of Massachusetts Chan Medical School, Worcester, MA, United States

**Keywords:** *Cryptococcus*, fungal vaccine, CD4 T cell, acquired immunodeficiency syndrome, protein family, cross-protection

## Abstract

Meningitis due to the fungal pathogen *Cryptococcus neoformans* is estimated to cause nearly 200,000 deaths annually, mostly in resource-limited regions. We previously identified cryptococcal protein antigens which, when delivered in glucan particles, afford vaccine-mediated protection against an otherwise lethal infection. Many of these proteins exhibit significant homology to other similar cryptococcal proteins leading us to hypothesize that protection may be augmented by immunologic cross-reactivity to multiple members of a protein family. To examine the significance of protein cross-reactivity in vaccination, we utilized strains of *Cryptococcus* that are genetically deficient in select antigens, yet are still lethal in mice. Vaccination with a protein without homologs (e.g., Mep1 and Lhc1) protected against challenge with wild-type *Cryptococcus*, but not against a deletion strain lacking that protein. Contrastingly, vaccination with a single chitin deacetylase (Cda) protein protected against the corresponding deletion strain, presumably due to host recognition of one or more other family members still expressed in this strain. Vaccination with a single Cda protein induced cross-reactive antibody and interferon-gamma (IFNγ) immune responses to other Cda protein family members. Paradoxically, we saw no evidence of cross-protection within the carboxypeptidase family of proteins. Factors such as *in vivo* protein expression and the degree of homology across the family could inform the extent to which vaccine-mediated immunity is amplified. Together, these data suggest a role for prioritizing protein families in fungal vaccine design: increasing the number of immune targets generated by a single antigen may improve efficacy while diminishing the risk of vaccine-resistant strains arising from gene mutations.

## Introduction

Fungal infections are a significant burden to public health. Despite causing an estimated 1.5 million deaths each year, there are currently no licensed antifungal vaccines (1). One such clinically important fungal disease is cryptococcosis, with *Cryptococcus neoformans* and its sister species *Cryptococcus gattii* being the main etiologic agents. Infection occurs predominately in immunocompromised populations, and patients can present with pneumonia-like symptoms following inhalation and subsequent infection of the yeast in the lungs. Disease is often undiagnosed until dissemination to the central nervous system (CNS) and the onset of cryptococcal meningitis (CM). As the cause of nearly 200,000 deaths annually, CM is responsible for approximately 15% of AIDS-related mortalities. Incidences of CM are highest in sub-Saharan Africa where upwards of 25 million inhabitants are fighting AIDS (2). Globally, CM in persons with AIDS has a case fatality rate upwards of 70%. While the fatality rate is lower in those who receive appropriate treatment, global mortality is skewed as disease severity is compounded by the limited access to treatment in resource-poor regions (2). Susceptible groups also include recipients of solid organ transplants and individuals with other immunodeficiencies. Development of a vaccine which protects vulnerable populations against cryptococcal infection would contribute greatly to diminishing disparities in global health.

While no cryptococcal vaccines have yet made it to human clinical trials, vaccines which protect mice from experimental cryptococcosis have been described [reviewed by Ueno et al. (3) and Oliveira et al. (4)]. One particularly prominent strategy is whole cell vaccination with live-attenuated and heat-killed cryptococcal mutants. For example, deletion of three cryptococcal chitin deacetylases (Cda) yields a chitosan-deficient, avirulent strain of *C. neoformans*: *cda1*Δ*2*Δ*3*Δ (*cda123*) (5). *cda123* is rapidly cleared from the lungs following inoculation and selectively enriches for protective Th1 populations (5). Significant protection is also generated by deletion strains *fbp1*Δ (6) and *sgl1*Δ (7), which induce protection by promoting differentiation of lung-infiltrating monocytes to monocyte-derived dendritic cells, and through the accumulation of immunomodulatory sterylglucosides, respectively. Others have generated mutants derived from *Cryptococcus* engineered to express particular gene products: P*_GDP1_-ZNF2* overexpresses the transcription factor which regulates the yeast to hyphae transition (8), and H99γ produces murine IFNγ (9). Each of these vaccine constructs demonstrate significant protection against a subsequent lethal challenge with a hypervirulent *C. neoformans* strain (5–9).

Hypothetical concerns to using whole organism vaccines include reactogenicity, triggering of autoimmunity, and, for live vaccines, the potential to cause disease in the immunocompromised population who are in the most need of a cryptococcal vaccine. Subunit vaccines obviate or mitigate these concerns. In an effort to develop subunit vaccines, we screened twenty-two recombinant cryptococcal protein antigens delivered in glucan particles (GPs) for the ability to protect mice against an otherwise lethal challenge with *C. neoformans* (10–12). These extracellular antigens were selected and tested for their previously demonstrated immunogenicity, their presence in protective alkaline extracts of *C. neoformans* (13), and/or their RNA transcript abundance in the cerebrospinal fluid of human patients (14). During down-selection of potential vaccine candidates, we considered several additional favorable criteria, such as orthologous genes in *C. gattii* and minimal homology to human proteins. We also speculated that antigens belonging to cryptococcal protein families might elicit cross-protective responses through their family members. Thirteen proteins from nine different families in addition to nine proteins lacking homologs (hereby referred to as “unique”) were represented in our screen. Analysis of the protective candidates revealed that most of them shared significant homology to other proteins within the *C. neoformans* proteome (nine of eleven), while most of the ineffective candidates lacked homologs (seven of eleven) (11). As such, we theorized that the potential for protein cross-reactivity with homologous proteins is beneficial for vaccine protection. If so, this would lend a rationale for prioritizing protein family antigens for experimental subunit vaccines.

For the present studies, we sought to determine whether homology across a protein family enhances vaccine efficacy by promoting a cross-reactive immune response. Four of the previously screened antigens belong to the chitin deacetylase family of proteins; three of these four are protective in our vaccine model: Cda1, Cda2, and Cda3 protect mice against *C. neoformans* challenge, while Fpd1 [sometimes referred to as Cda4 (15)] does not (10–12). Another protective antigen, Cpd1, belongs to a family of three carboxypeptidases (Cpd) with extensive sequence conservation (11). Contrastingly, Mep1, a metalloprotease, is a protective vaccine antigen that lacks homologs within the *C. neoformans* proteome (11). An additional protein without homologs, lactonohydrolase Lhc1 (16), also protects mice; the data on the GP-Lhc1 vaccine are reported here for the first time. By comparing the efficacy of vaccines containing Cda and Cpd protein family members with vaccines containing Mep1 and Lhc1 (which lack homologs), we studied whether intra-family cross-reactivity can improve protection. In addition to our standard model of experimental cryptococcosis using the reference *C. neoformans* strain KN99α, we tested genetic deletion strains of *Cryptococcus* missing vaccine antigens, but which retained virulence. Finally, we performed *ex vivo* studies examining adaptive cross-reactive humoral and cell-mediated immune responses in mice vaccinated with Cda family members.

## Materials and methods

### Chemicals and culture media

Reagents were purchased from Thermo Fisher Scientific (Pittsburgh, PA, USA), unless otherwise stated. *Cryptococcus* strains were cultured in YPD (Difco yeast extract, Bacto peptone, 2% dextrose, with and without agar) and on Sabouraud dextrose agar. *E. coli* strains TOP10 and BL21 were cultured at 37°C in LB broth or agar. Transformed strains of BL21 were used for protein expression by being cultured for 18 h at 30°C with shaking in Overnight Express Instant TB medium (MilliporeSigma, Burlington, MA, USA). Antibiotic selection was with ampicillin (100 μg/ml) or kanamycin (100 μg/ml).

### Strains of *Cryptococcus*

*C. neoformans* var. *grubii* strains (**Table 1**) were maintained as glycerol stocks at -80°C. Strain KN99α was used as the wild-type (WT) strain in these studies, and is isogenic to strain H99, which was used to derive the deletion strains (17). Initial cultures were grown on YPD agar, and, for *in vivo* challenge, strains were cultured in liquid YPD at 30°C with shaking for ~18 hours. Cells were harvested by centrifugation and washed twice in phosphate buffered saline (PBS). Cell counts were obtained using the TC20 automated cell counter (BioRad, Hercules, CA, USA), and the strains were suspended in PBS at concentrations of 4x10^5^ colony forming units (CFU)/mL. CFU of the inoculum was verified by plating on Sabouraud dextrose agar.

**Table 1:** *C. neoformans* strains used.

Strain	CNAG^1^	Protein^2^	Accession #^3^	Source for *Cryptococcus* strain
KN99α^4^	n.a.	n.a.	n.a.	Janbon et al., 2014 (17)
*cda1*Δ	05799	Cda1	XP_012050538.1	Baker et al., 2007 (18)
*cda2*Δ	01230	Cda2	XP_012049402.1	Baker et al., 2007 (18)
*cda3*Δ	01239	Cda3	XP_012049409.1	Baker et al., 2007 (18)
*cpd1*Δ	00919	Cpd1	XP_012049193.1	FGSC, Madhani Deletion Collection 2015, plate 4, well A12
*mep1*Δ	04735	Mep1	XP_012051981.1	FGSC, Madhani Deletion Collection 2015, plate 6, well H9
*lhc1*Δ	04753	Lhc1	XP_012051973.1	FGSC, Madhani Deletion Collection 2015: plate 0, well E6

^1^CNAG numbers are from the NCBI database for *C. neoformans* var. *grubii* strain H99 (taxid:235443).

^2^Protein missing from the deletion strain.

^3^Accession # refers to the NCBI protein accession number.

^4^KN99α is derived from strain H99 and is used as the wild-type *C. neoformans* strain in these studies.

n.a., Not applicable; FGSC, Fungal Genetics Stock Center.

### Expression of cryptococcal proteins in *E. coli* and protein purification

Ovalbumin (Ova) and Lhc1 were synthesized and cloned in pET19b by Genscript (Piscataway, NJ, USA). Recombinant proteins were expressed in *E. coli* and purified using His·Bind resin (MilliporeSigma) as previously described (10). Briefly, BL21 *E. coli* containing recombinant protein-expressing plasmid were inoculated into Overnight Express Instant TB medium (MilliporeSigma) from glycerol stocks maintained at -80°C. Cells were lysed and soluble material was purified over a His·Bind nickel column, with all purification buffers containing 6M urea to maintain protein solubility. Eluted fractions were selected based on SDS-PAGE analysis and pooled for dialysis to remove imidazole. Dialyzed protein was concentrated to 10 mg/mL, and protein stocks were stored at -80°C.

### Vaccines

Glucan particle (GP) vaccines were prepared as previously described with each dose containing 10 μg of antigen (10, 11, 13). Cationic adjuvant formulation 01 (CAF01) vaccines (19–21) were formulated such that each dose contained 5 μg of antigen in 0.5 μL combined with 49.5 μL of 10 mM Tris/2% glycerol (pH 7.0) and 50 μL CAF01. CAF01 was obtained from Statens Serum Institut, Copenhagen, Denmark and used under the terms of a Material Transfer Agreement.

### Mice

BALB/c (Strain #000651), C57BL/6 (Strain #000664), muMT (Strain #002288), B2m (Strain #002087), and MHCII (Strain #003584) mice of both sexes were purchased from the Jackson Laboratory (Bar Harbor, ME, USA). Mice were housed and bred in a specific pathogen-free environment at the University of Massachusetts Chan Medical School (UMCMS). All experimental protocols were approved by the UMCMS Institutional Use and Care of Animals Committee.

### Vaccination and challenge

For both GP-vaccines and CAF01 vaccines, mice received a series of three vaccinations administered as subcutaneous injections of 0.1mL per dose in the abdomen, with two weeks between vaccinations. Two weeks following the final vaccination, mice were anesthetized with isoflurane and challenged by orotracheal infusion into the lung with 2x10^4^ CFU of the indicated *C. neoformans* strain in 50 μL PBS. Survival was monitored until 70 days post infection (DPI), at which point survivors were euthanized.

### Immunoblots

Sera were collected via cardiac puncture two weeks following the third vaccination and pooled from 4 mice/vaccination group. Recombinant protein (2 μg per lane) was electrophoresed into a 4-20% gradient gel (BioRad) in Tris-glycine-SDS running buffer. Protein standards were from BioRad. The reference gel was stained with Coomassie InstantBlue (Abcam, Cambridge, United Kingdom) and replicate gels were transferred to nitrocellulose membranes (BioRad) using a BioRad Trans-Blot Turbo Transfer System. Blots were blocked with EveryBlot Blocking Buffer (BioRad), incubated with pooled serum at a 1:500 dilution followed by goat anti-mouse IgG (H+L) conjugated with alkaline phosphatase (Invitrogen) as the secondary antibody at a 1:5,000 dilution. Blots were developed and visualized using 1-Step NBT/BCIP.

### *Ex vivo* stimulation of splenocytes

Spleens were harvested two weeks after the third vaccination. Single cell suspensions were prepared by pressing each spleen through a 70 micron screen (MTC Bio, Sayreville, NJ). Following red blood cell lysis with 1X RBC Lysis Buffer, splenocytes were washed twice with RPMI supplemented with 10% fetal bovine serum (FBS). Cell counts were obtained using the TC20 automated cell counter, and live and dead cells were differentiated using Trypan Blue (BioRad). Cells were then suspended at a concentration of 10^7^ live cells/mL in complete RPMI (10% FBS, 1% GlutaMax, 1% HEPES, 100 U/mL penicillin, and 100 μg/mL streptomycin). Splenocytes at 5x10^6^/mL, unless otherwise noted were seeded into tissue culture-treated, round-bottom 96 well plates at a final volume of 200 μL. Cells were stimulated with recombinant protein at a final concentration 5 μg/mL. Duplicate wells were done for each stimulus. Culture supernatants were collected after three days incubation at 37°C, and mouse IFNγ levels were measured by ELISA according to the manufacturer specifications (R&D Systems, Minneapolis, MN, USA).

### Bioinformatics

Protein sequences for *C. neoformans* var. *grubii* strain H99 (taxid:235443), were obtained using their respective CNAG numbers from the National Center for Biotechnology Information (NCBI) protein database. Protein homologies were quantified using Basic Local Alignment Search Tool protein (BLASTP) (22) and multiple sequence alignments were generated using MUSCLE (3.8) (23). RNA seq data was downloaded from Gene Expression Omnibus (24).

### Statistics

Graph generation and statistical analyses were conducted using GraphPad Prism V 8.1.1 (GraphPad Software, San Diego, CA, USA). The Mantel-Cox, log-rank test was used to compare Kaplan-Meier survival curves. P-values for survival studies were determined by pair-wise comparisons, and the Bonferroni correction was applied to adjust for multiple comparisons before statistical significance was denoted. Significance of *ex vivo* studies measuring IFNγ were determined by one-way ANOVA with Welch’s correction.

## Results

**Figure 1:**
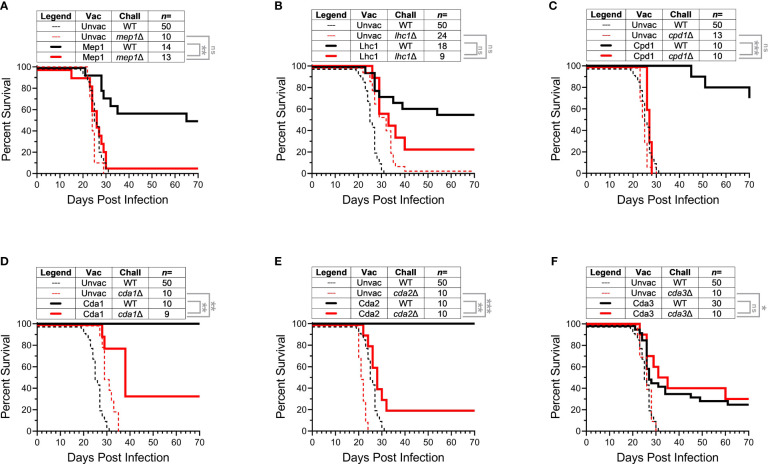
Infection with *Cryptococcus* strains deleted of protein used for vaccination. BALB/c mice received three subcutaneous GP-recombinant protein vaccines containing 10 μg antigen/dose before they were challenged with 2x10^4^ CFU of the indicated strain of *Cryptococcus neoformans*. Survival was measured until 70 DPI. For each group, data were combined from a minimum of two independent experiments. Vaccine antigens either had no cryptococcal homologs **(A, B),** belonged to the Cpd protein family **(C)**, or the Cda protein family **(D-F)**. *C. neoformans* KN99 was used as the wild-type strain (WT) and compared to single gene deletion strains corresponding to each studies’ vaccine protein. The number of mice per group is indicated by *n*=. Kaplan-Meier survival curves were compared using the Mantel-Cox, log-rank test. To adjust for multiple comparisons, significance was determined after applying the Bonferroni correction. ns, not significant; * significant at P ≤ 0.0125; ** significant at P ≤ 0.0025; *** significant at P ≤ 0.00025.

For these studies, we utilized strains of *C. neoformans* genetically deficient in select vaccine antigens (**Table 1**). The vaccines protected BALB/c mice from WT *C. neoformans* KN99 challenge, but would mice remain protected if the challenge strain did not express the protein used to confer immunity? In addition, the corresponding deletion strains, while in some cases reduced in virulence, remained lethal in unvaccinated mice. To establish our model, we first examined protective vaccines lacking homologs within the *C*. *neoformans* genome. Mep1 is a secreted metalloprotease which was shown to be abundantly transcribed during human infection (14, 25). Vaccination with GP-Mep1 yielded ~50% survival following challenge with *C. neoformans* KN99 (11). As Mep1 has no cryptococcal homologs, we predicted the vaccine would be ineffective in mice challenged with a *C. neoformans* strain lacking Mep1. We found that mice vaccinated with GP-Mep1 and challenged with *mep1*Δ survived no differently than unvaccinated mice challenged with *mep1*Δ (**Figure 1A**). Thus, GP-Mep1 vaccinated mice were protected only when the challenge strain expressed Mep1.

Lhc1 also lacks cryptococcal homologs. Lhc1 is a capsule-associated lactonohydrolase (16) which has not been previously tested as a candidate vaccine antigen. Vaccination with GP-Lhc1 protected BALB/c mice against *C. neoformans* KN99 challenge **(** **Figure 1B** **).** Over half of the vaccinated mice survived until the termination of the experiment at 70 DPI. The cryptococcal deletion strain missing this vaccine antigen, *lhc1*Δ, has been shown to be slightly attenuated *in vivo* compared to KN99 (16). Indeed, we saw that *lhc1*Δ challenge in unvaccinated mice resulted in delayed death compared to mice challenged with KN99 (**Figure 1B**). Importantly though, vaccination with GP-Lhc1 did not significantly protect mice from challenge with *lhc1*Δ: there was no statistical difference in survival for mice challenged with *lhc1*Δ regardless of whether they were vaccinated with GP-Lhc1 or left unvaccinated (**Figure 1B**). As vaccination in this case did not significantly change the survival outcome, we interpreted the curve as showing a loss of vaccine-mediated protection. However, the statistics denoted that (unlike with Mep1) there is no statistical difference between vaccinated groups challenged with either the WT or the deletion strain. The trend was that survival was decreased in GP-Lhc1 vaccinated mice challenged with *lhc1*Δ compared to those that received the WT challenge, though the difference was not as robust as what we saw in the case of Mep1 (**Figures 1A, B** **)**. We attribute this to the documented attenuated virulence of *lhc1*Δ (16). Taken together, the data with Mep1 and Lhc1 demonstrate that protection is lost if the vaccine antigen is absent from the challenge strain.

We next investigated whether cross-protection induced by homologous antigens provides a survival benefit in vaccinated mice. Cpd1 belongs to the carboxypeptidase family of proteins; two other members of this family, Cpd2 and Cpd3, share significant homology to Cpd1 (**Figure S1**). We questioned whether GP-Cpd1 vaccination would still yield protection against a strain deficient in Cpd1 (*cpd1*Δ). *cpd1*Δ is a deletion strain specifically targeting Cpd1, and not its homologs. We reasoned the sequence similarity of Cpd2 and Cpd3 to vaccine antigen Cpd1 could trigger immune recognition and induce a protective, vaccine-mediated response to fungal challenge even in the absence of Cpd1. In agreement with our previous work (11), GP-Cpd1 vaccination generated robust protection against *C. neoformans* KN99 challenge (**Figure 1C**). However, we saw no difference in survival between vaccinated and unvaccinated groups challenged with *cpd1*Δ. Thus, there was no evidence of cross-protection induced by the GP-Cpd1 vaccine (**Figure 1C**).

While Cpd1 is a protective vaccine antigen, we have not yet investigated whether its homologs are also protective. The Cda family, however, is more thoroughly characterized in our model, and consists of four proteins (Cda1, Cda2, Cda3, and Fpd1) (**Figure S2**). When formulated in GP-based vaccines, Cda1, Cda2, and Cda3 protect mice against challenge with WT *C. neoformans* strains (10, 11). Additionally, mice challenged with *cda2*Δ still generate Cda2-MHCII tetramer+ CD4^+^ T cells in the lungs, albeit significantly less pronounced than that seen with WT KN99 challenge; this population was attributed to the tetramer likely binding closely related cryptococcal proteins (26). For these reasons, we anticipated that the Cda family of homologous proteins would have increased likelihood of inducing cross-protection.

GP-Cda1 and GP-Cda2 are the more protective vaccines of the Cda family (11), and both conferred 100% survival in vaccinated mice challenged with WT KN99 (**Figures 1D, E** **)**. We found GP-Cda1 vaccination followed by challenge with *cda1*Δ resulted in a survival curve which fell between vaccinated, KN99 challenged and unvaccinated, *cda1*Δ challenged (**Figure 1D**). The same pattern occurred for GP-Cda2 (**Figure 1E**). Protection was not as robust as when the vaccine antigen was deleted in the challenge strain, but there was a significant benefit over unvaccinated groups. With GP-Cda3, we found that vaccinated mice had similar survival regardless of whether they were challenged with WT KN99 or *cda3*Δ. Moreover, for both groups survival was significantly improved over the unvaccinated groups (**Figure 1F**).

**Figure 2:**
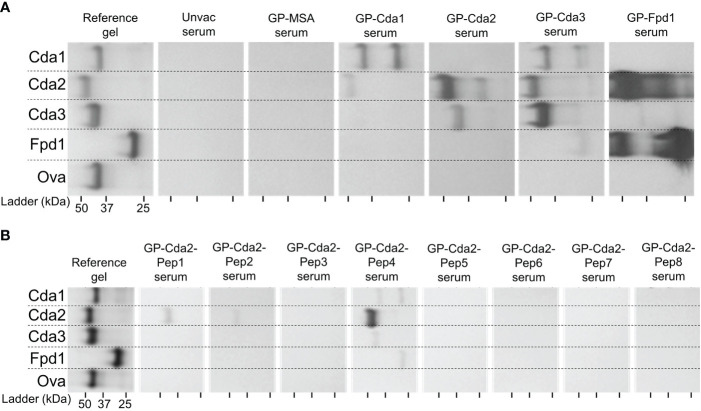
Vaccination with any of the Cda family proteins induces serum IgG reactive with at least one other family protein. BALB/c mice were vaccinated as in **Figure 1**, and mice were euthanized for serum collection two weeks after the final vaccination. The reference gel shows the SDS-PAGE of purified recombinant proteins used in GP-vaccines. Each lane contains 2 μg of protein, and the gels were stained with Coomassie. To the right are the Western Blots against serum from mice treated with the GP-vaccines. Serum from mice was applied as a primary antibody with α-mouse IgG as the secondary. Serum was pooled from groups of n=4 for the assay. Mice vaccinated with GPs loaded with mouse serum albumin (MSA) served as a negative vaccine control, and recombinant ovalbumin (Ova) was used as a protein control. Blots are oriented such that the top of the gel is on the left, and molecular masses of the protein standards are denoted by the ticks on the bottom such that the left, middle, and right ticks for each blot represent a protein ladder of 50 kDa, 37 kDa, and 25 kDa, respectively. **(A)** Serum from mice vaccinated with the indicated recombinant protein. **(B)** Serum from mice vaccinated with the indicated synthesized Cda2 peptide. See Specht et al., 2022 (12) and **Figure S3** for the peptide sequences. The Western blots have been rotated and cropped to show the relevant portions but not spliced or digitally manipulated.

Having demonstrated a cross-protective response between members of the Cda family, we next looked for immunologic cross-reactivity by conducting *ex vivo* assays measuring antibody and cytokine production. We first probed for cross-reactive IgG responses within the Cda family. BALB/c mice were vaccinated with GPs formulated with either Cda1, Cda2, Cda3, or Fpd1. Mice vaccinated with GPs loaded with mouse serum albumin (MSA) and unvaccinated mice were used as controls. Two weeks after the third vaccination, serum was collected and analyzed by immunoblot for antibodies against recombinant proteins. As expected, IgG responses to the Cda family proteins were not detected in GP-MSA and unvaccinated mice. Vaccination with any of the Cda proteins elicited an IgG response to that respective protein (for example, Cda1 vaccination induced IgG which binds to Cda1) (**Figure 2A**). Additionally, for each of these four vaccines, serum reacted with at least one other protein family member. Thus, in addition to binding Cda1 protein, vaccination with GP-Cda1 also stimulated antibody cross-reactive to Cda2. GP-Cda2 immune serum contained IgG, which also bound Cda3; GP-Cda3 immune serum bound Cda1, Cda2, and Fpd1; and GP-Fpd1 immune serum bound Cda2 and Cda3 (**Figure 2A**). Because our antigens are expressed as His-tagged proteins in *E. coli*, as an additional control, we expressed and purified recombinant Ova protein following the same protocol used for the tested cryptococcal protein antigens. None of the sera from vaccinated mice bound to the Ova control.

We have previously shown peptides derived from Cda2 protect BALB/c mice when used in GP-based vaccines (12). To gain further insight into which regions of the proteins may be involved in cross-reactive antibody responses, we probed the serum from mice vaccinated with GP-Cda2-peptides against recombinant proteins from the Cda family. Of the eight peptide vaccines tested, GP-Cda2-Pep1, GP-Cda2-Pep2, and GP-Cda2-Pep4 stimulated IgG which bound Cda2 protein on immunoblots (**Figure 2B**). Serum from GP-Cda2-Pep4 vaccinated mice had the most robust response and also had cross-reactivity with the other Cda family proteins. As potential background arising from contaminating *E. coli* proteins and/or shared His tags in recombinant protein vaccines is not a factor with synthesized peptide vaccines, none of the sera bound the recombinant Ova control.

**Figure 3:**
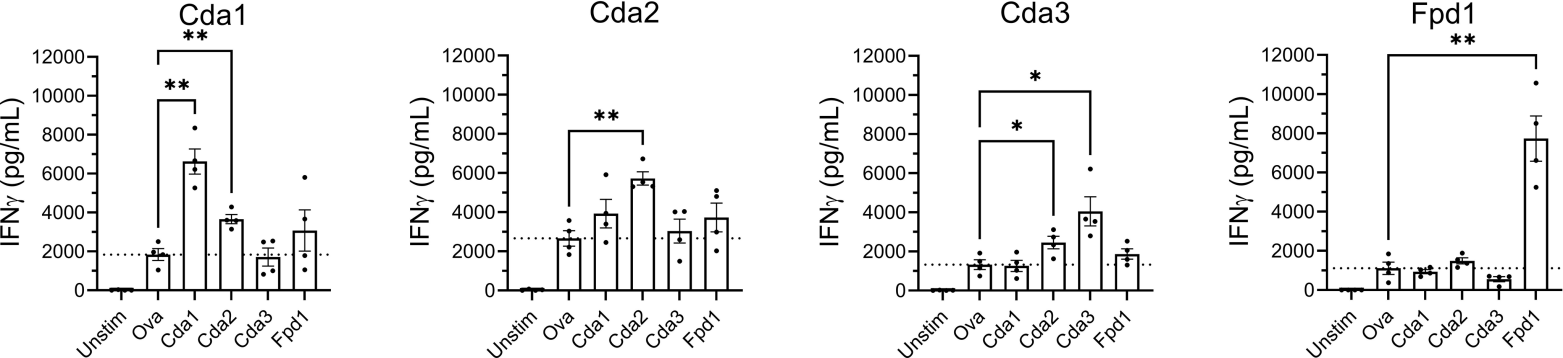
*Ex vivo* stimulation of splenocytes from mice vaccinated with Cda proteins induce an IFNγ response to other protein family members. For Cda family proteins, BALB/c mice were vaccinated 3 times subcutaneously with 5 μg of antigen/dose adjuvanted with CAF01. Two weeks after the final vaccination, splenocytes were prepared from harvested spleens and 10^6^ cells/well left unstimulated (Unstim), or cultured with the indicated recombinant protein as denoted under the X axis. After 3 days of culture, the supernatants were analyzed for IFNγ by ELISA. Data are expressed as means ± the standard error of the mean. N=4 mice/group; each dot represents the average of two technical replicates for a single mouse. The dotted horizontal line represents the mean IFNγ production of cells stimulated with Ova. Recombinant Ova was expressed in *E. coli* and purified following the same protocol as recombinant cryptococcal proteins. Significance was determined by one-way ANOVA with Welch’s correction; comparisons were made to Ova. * significant at P ≤ 0.01; ** significant at P ≤ 0.001.

The observed IgG responses demonstrate the ability for vaccination with a single Cda protein to induce cross-reactivity to other family members. As IFNγ is required for effective antifungal responses to *Cryptococcus* (27–29), we tested the ability of splenocytes from Cda-vaccinated mice to produce IFNγ following *ex vivo* stimulation with each of the individual Cda family proteins. Two weeks after the final vaccination, mice were euthanized following which splenocytes were prepared and cultured for three days with various stimuli (**Figure 3**). For all Cda-vaccinated groups, unstimulated cells produced nearly undetectable levels of IFNγ. As with the Western blots, we utilized recombinant Ova protein to control for potential impurities remaining after recombinant protein expression in *E. coli*. The levels of IFNγ seen after stimulation with Ova was set as the background and indicated as dotted horizontal lines in **Figure 3**. For each group, stimulation with the original vaccine antigen resulted in significant IFNγ production (**Figure 3**). For example, splenocytes from the mice vaccinated with Cda1 produced IFNγ in response to stimulation with Cda1 protein. We also found evidence of a cross-reactive IFNγ response for two of the Cda family antigens as cells from Cda1 and Cda3 vaccinated mice made IFNγ in response to Cda2 stimulation (**Figure 3**).

**Figure 4:**
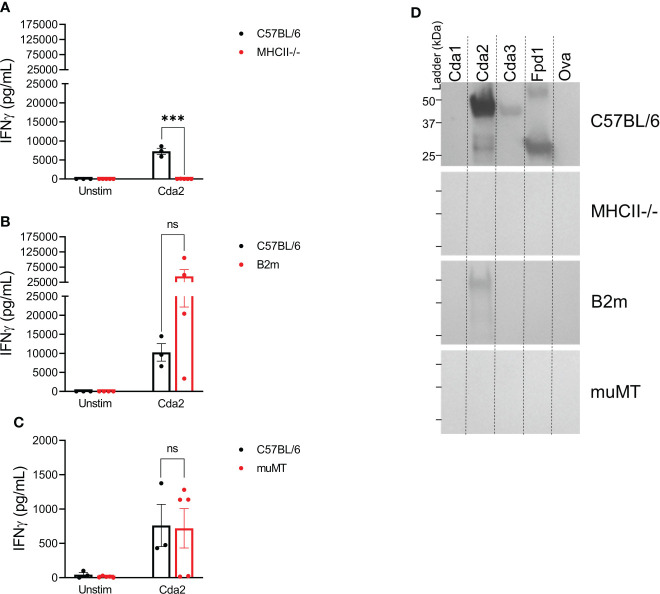
Loss of CD4^+^ T cells ablates serum IgG and *ex vivo* IFNγ production in the splenocytes of GP-Cda2 vaccinated mice. Mice were vaccinated as in **Figure 2**. Two weeks after the third vaccination, the mice were euthanized and spleens and serum were collected for analysis as in **Figure 3** and **Figure 2**, respectively. **(A–C)** IFNγ production by the splenocytes of GP-Cda2 vaccinated mice following *ex vivo* stimulation. Error bars represent the standard error of the mean, with each sample graphed as the average of 2 technical replicates. Significance was determined by ordinary one-way ANOVA with Welch’s correction. ns, not significant; or *** significant at P ≤ 0.0001. **(A)** n=3 mice for C57BL/6 and n=5 mice for the MHCII-/-. **(B)** n=3 mice for C57BL/6 and n=4 for B2m. **(C)** Due to the small size of spleens from muMT mice, only 2.5x10^5^ splenocytes were stimulated per well. C57BL/6 n=3 and muMT n=5. **(D)** Western blots for IgG response in the pooled serum of GP-Cda2 vaccinated mice. Molecular masses of the protein standards are denoted by the ticks on the left such that the top, middle, and bottom ticks for each blot are 50 kDa, 37 kDa, and 25 kDa, respectively. The Western blots have been cropped to show the relevant portions but not spliced or digitally manipulated.

We next examined the contributions of T cells and B cells to the cross-reactive IgG and IFNγ immune responses following vaccination with Cda2. WT C57BL/6 mice were compared with MHCII^-/-^ mice, which lack functional CD4^+^ T cells, B2m mice, which are deficient in CD8^+^ T cells, and muMT mice, which lack mature B cells; mutant mice are on a C57BL/6 background. Similar to what was seen with WT mice on the BALB/c background, *ex vivo* stimulation of the splenocytes of WT C57BL/6 mice showed a pronounced IFNγ response to Cda2 protein (**Figure 4A**). Splenocytes from MHCII^-/-^ mice, which are deficient in CD4^+^ T cells, however, produced nearly undetectable amounts of IFNγ with Cda2 stimulation (**Figure 4A**). We also compared splenocyte IFNγ production in B2m mice, which lack functional CD8^+^ T cells, to WT mice following stimulation with Cda2 protein. There was no significant difference in IFNγ production between these two groups, though the B2m group did trend higher than WT (**Figure 4B**). Similarly, the absence of B cells in muMT mice did not significantly inhibit IFNγ production in response to Cda2 in vaccinated mice (**Figure 4C**). Because muMT mice have much smaller spleens than WT mice (30, 31), we could not compare the response of muMT mice following our standard protocol using 10^6^ splenocytes per well for individual mice. To circumvent this issue, we assayed individual mice using only 2.5x10^5^ cells per well. The mean IFNγ production following stimulation with Cda2 was similar between WT and muMT mice under these conditions (**Figure 4C**). Although the lack of B cells did not significantly impact the ability of vaccinated animals to produce IFNγ, MHCII^-/-^ mice did not generate a detectable IgG antibody response to Cda2 or its homologs (**Figure 4D**) suggesting the importance of CD4^+^ T cell help in vaccine-induced antibody responses. B2m mice also exhibited defects in their IgG response; Cda2 vaccinated mice made IgG against Cda2, albeit at a lesser magnitude than seen with WT mice. Moreover, IgG cross-reactive with other Cda family members was not detected (**Figure 4D**). The decreased antibody response in B2m mice is not unexpected as it has been documented in other vaccine models (32, 33).

**Figure 5:**
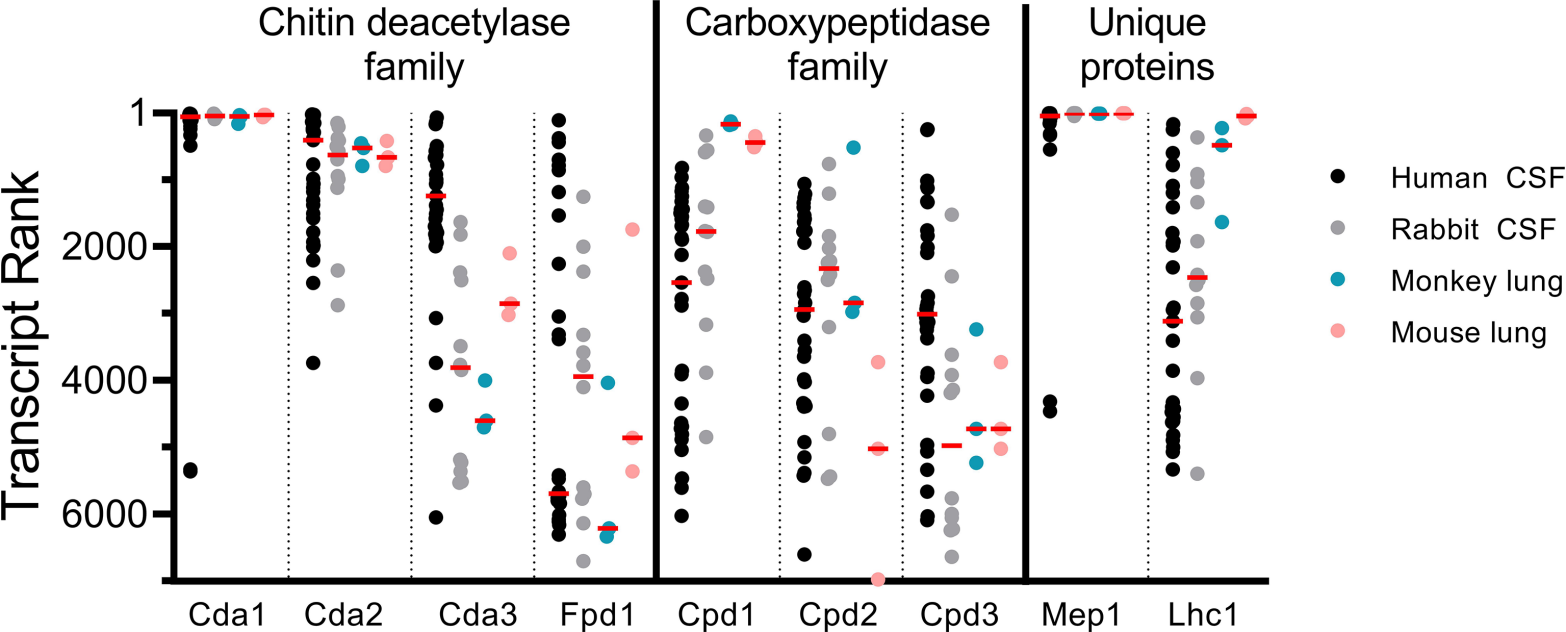
Vaccine antigen gene expression *in vivo*. Transcript rank is in accordance with the gene with the highest number of RNA seq reads being ranked “1”. Thus, the more abundantly transcribed genes are towards the top of the Y axis, while those with fewer transcripts are lower. RNA seq data are displayed for *C. neoformans* isolated from the CSF of human patients (n=33) (14, 34), the CSF of rabbits 1 day post intracisternal infection with *C. neoformans* clinical isolates (n=12) (34, 35), the lungs of monkeys 7 days after intratracheal infection with *C. neoformans* H99 (n=3) (36), and the lungs of C57BL/6 mice 7 days after intranasal infection with *C. neoformans* H99 (n=3) (36). Each dot represents an individual sample, except in the case of rabbit CSF, where each dot represents a pool of the CSF of 3 rabbits (n=12 pools of 3) (34, 35). Median values are denoted by the red horizontal bar. Transcripts from 6,967 genes were found in human CSF; 6,962 genes in rabbit CSF; and 6,975 genes in both monkey and mouse lungs (14, 34–36).

Antigen expression during infection is required to elicit protective and cross-protective immune responses. To gain insight into the relative expression of the vaccine antigens during cryptococcosis, we analyzed RNAseq data deposited into Gene Expression Omnibus (24). During our initial vaccine screen, abundant transcription during human infection was a criterion for selection of candidate antigens; however, this was based on samples from only two patients (11, 14). This data set has since been expanded to include RNAseq analysis of CSF samples from an additional 31 patients who presented with CM (34) (data available at NCBI GEO database (24), accession GSE171092). We sorted these transcripts from most to least abundant for each of our proteins of interest, such that a rank of “1” indicates the highest number of reads for that sample (**Figure 5**). This allowed us to assess variability of gene expression associated with vaccine antigens for clinical *C. neoformans* isolates. We compared these transcript rankings with gene expression rankings in published experimental models of cryptococcosis including CNS infection in immunocompromised rabbits, and pulmonary infections in monkeys and mice (14, 34–36) (data available at NCBI GEO database (24), accessions GSE171092, GSE136879, and GS122785). For the human CSF samples, there is much variability in gene expression as assessed by transcript rank. Nevertheless, transcript rank is more closely clustered for Cda1, Cda2, and Cda3, than for Cpd1, Cpd2, or Cpd3. Gene transcript rank also varied as a function of experimental model: Cda1, Cda2, and Mep1 expression is fairly consistent throughout human CSF and animal models of infection, while others, such as Lhc1 and Cda3, are considerably more heterologous (**Figure 5**). Despite small sample sizes, mouse and monkey lung data sets are comparable for most of the antigens for most transcript ranks. Both Cda1 and Cda2 are abundantly transcribed in each of the data sets examined. Fpd1, which is the only non-protective member of the Cda family, however, ranks much lower across samples. The median transcript rank for Cpd1, Cpd2, and Cpd3 does not vary greatly in human CSF. In the monkey and mouse lung, however, Cpd1 transcript is at a much higher level than Cpd2 or Cpd3 (**Figure 5**).

## Discussion

**Figure 6:**
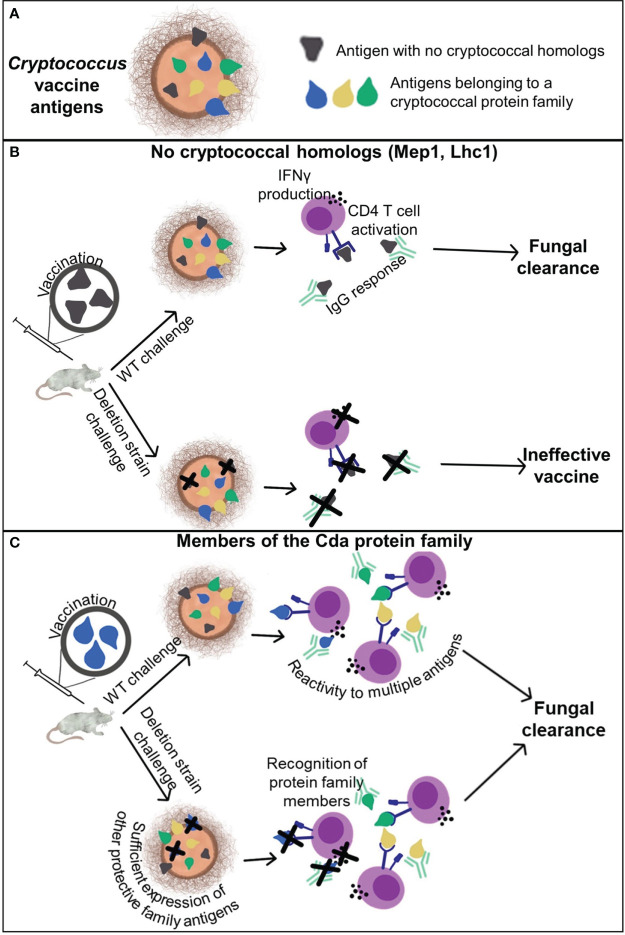
Cross-reactivity and cross-protection generated by cryptococcal vaccination. **(A)** Cryptococcal antigens can be categorized by those that have no homologs within the genome and those belonging to a protein family. **(B)** Antigens which do not have any homologs are protected by the adaptive immune response to the respective recombinant protein vaccine only when the antigen is present in the challenge strain. **(C)** Vaccination with a single antigen that belongs to a protein family, such as the Cda family, can elicit protective adaptive immune responses to the vaccine antigen as well as other antigens within the family. This enables vaccine-mediated protection against deletion strains of *Cryptococcus* lacking the vaccine antigen.

These experiments aimed to determine the contribution of cross-reactivity within protein families to vaccine-mediated protection. Key to understanding the potential for cross-protection, two protein antigens (Mep1 and Lhc1) without homologs were also studied. We confirmed that vaccination with these unique protein antigens does not protect mice from infection by a strain of *Cryptococcus* that does not express the antigen (**Figure 6**). We were unable to demonstrate cross-protection within the Cpd family of proteins, but we did find that vaccination with a Cda family protein can provide a survival benefit against challenge with the corresponding deletion strain (**Figure 6**). Our subsequent studies then focused on the three previously identified protective vaccine antigens belonging to the Cda family of proteins (10, 11). We found that homology to other cryptococcal antigens is necessary, but is not always sufficient, to generate cross-protection. This could in part be attributed to a cross-reactive IgG response to other protein family members found *in vivo*. More importantly, as IFNγ is critical for protection against cryptococcal infection (27), we saw a cross-reactive IFNγ response to Cda proteins in *ex vivo* splenocyte stimulation assays. These data support the prioritization of protein families in subunit vaccine design and implicate identifiable factors that should be considered for generating cross-protection.

The reasons for the lack of cross-protection following Cpd1 vaccination and *cpd1*Δ challenge are speculative. Cpd2 and Cpd3 share 77% and 68% sequence identity with the full-length Cpd1 protein, respectively. However, the protective epitope(s) embedded in the Cpd1 protein sequence are unknown. A cross-reactive immune response would be expected only if these epitope(s) are shared with Cpd2 and Cpd3. An additional consideration is the relative availability of the Cpd1, Cpd2, and Cpd3 antigens during *in vivo* infection, as the pulmonary compartment is the initial site of infection, Most relevant to these studies is the RNAseq data of infected mouse lungs shows the median transcript rank of Cpd1 to be over ten times higher than Cpd2 or Cpd3. The study of cryptococcal transcripts in both the lungs and CSF is relevant clinically because exposure is thought to occur most commonly as a result of inhalation into the lungs, following which dissemination to the CNS occurs if pulmonary defenses fail. Thus, vaccine antigens should ideally be expressed in both the lungs and CNS. A similar trend was observed in samples from the lungs of monkeys, wherein Cpd1 transcripts were much more abundant than Cpd2 or Cpd3 transcripts. What we can extrapolate from these available data sets is limited in that mRNA abundance does not necessarily correlate with protein production. Analysis by mass spectrometry of extracts from acapsular *C. neoformans* grown in culture identified Cpd1 as the seventh most abundant protein whereas Cpd2 and Cpd3 were not detected (13). Caveats regarding the proteomic data include that the extracts were from cultured cells, not *in vivo* organisms, and the analysis did not examine proteins secreted into the culture medium. Vaccination studies using Cpd2 and Cpd3 vaccines could provide further mechanistic insights.

Unlike with Cpd1, vaccines containing Cda1, Cda2, or Cda3 still significantly protect mice even if the vaccine antigen is removed from the challenge strain. Nevertheless, for the vaccines containing Cda1 and Cda2, protection against the deletion strain is not as robust when compared to the WT strain. This suggests that immune recognition of the homologous antigens provides cross-protection that is able to partially, but not completely, compensate for loss of the primary antigen. Interestingly though, vaccination with GP-Cda3 protected mice challenged with either WT KN99 or *cda3*Δ to a similar extent. This suggests that protection generated by Cda3 vaccination is due to cross-reactivity to either Cda1 or Cda2. Future experiments detailing mechanisms of protection will be informative as to whether there is a dominant antigen within the Cda family.

Members of the Cda family each develop an IgG response to at least one other protein within the family. While the importance of antibody in mediating protection in our model is still undefined, these experiments did provide the foundation for the possibility of cross-reactivity. We were then able to observe cross-reactivity to these family antigens in a more physiologically relevant context: Increased IFNγ is strongly associated with improved clinical outcomes in patients with CM (27). *Ex vivo* stimulation of splenocytes from vaccinated mice show that vaccination with a single Cda protein can induce an IFNγ response to multiple proteins within the family. Splenocytes from mice vaccinated with Cda1 and with Cda3 produced IFNγ in response to the respective vaccine antigen, but also in response to Cda2. Vaccination with Fpd1, which is the only non-protective antigen in the Cda family, did not result in IFNγ production in response to any stimulus other than itself. As mentioned in the context of survival, this lends to the idea of a dominant antigen, likely Cda1 or Cda2, within the family mediating protection. Not only are Cda1 and Cda2 abundantly transcribed in both human CSF and in the mouse lung, proteomics also revealed Cda2 to be an abundant antigen in cryptococcal extracts (13). It is important to note, however, that we have not yet established the impact of glycosylation in our vaccine model, nor its role in cross-reactivity. Our vaccine antigens were generated such that serine/threonine-rich regions were removed from the sequence before expression in *E. coli*; in fungal proteins, these are often areas of O-glycosylation, but *E. coli*-derived proteins are not glycosylated. Cda2 is a cryptococcal protein which, in its native form, is heavily glycosylated (37). Vaccination with non-glycosylated Cda2 is very protective against *C. neoformans* challenge, but we have also shown that recognition of glycosylated regions of Cda2 by the mannose receptor induces a stronger CD4+ T cell response than that seen with non-glycosylated Cda2 (37). It is possible that the lack of glycosylation of Cda family proteins used in vaccination may affect the extent of cross-protection within this family.

Having established cross-reactivity within the Cda family, we were curious as to the cells driving these responses. To address this, we vaccinated mice deficient in specific lymphocyte subpopulations. GP-Cda2 vaccinated mice deficient in CD4^+^ T cells do not produce IFNγ in response to *ex vivo* stimulation with the vaccine antigen. Deficiency in CD8^+^ T cells or mature B cells, however, did not have a deleterious effect on IFNγ levels in culture supernatants. This suggests that CD4^+^ T cells are major contributors to the antigen-specific IFNγ response. As individuals with CD4^+^ T cell deficiencies are at increased risk for cryptococcal infections, the potential clinical impact of these observations is unknown. The MHCII^-/-^ mice used in these studies have a complete CD4^+^ T cell deficiency, but the CD4^+^ T cell levels of patients with cryptococcal disease are variable. We have not determined whether there is a CD4^+^ T cell threshold for retention of vaccine efficacy, or whether this threshold could be shifted with adjunctive IFNγ therapy (38). Ongoing studies examining CD4^+^ T cell responses at the site of infection and the contribution of other cell types that produce IFNγ should further our mechanistic understanding of vaccine-mediated protection.

Historically, one strategy for prioritizing vaccine antigens has been to target those which are essential to either survival or virulence. This decreases the chances of selective pressure promoting the generation of deletion mutants, as this will result in non-viable or significantly attenuated strains. The implications of selecting vaccine targets, which are not essential to the pathogen can be seen with *Bordetella pertussis*, the causative agent of whooping cough. The acellular pertussis (aP) vaccine administered to children is composed of several *B. pertussis* proteins, with many formulations containing the antigen pertactin (PRN) (39). Following the replacement of the whole cell pertussis vaccines with aP, PRN-deficient strains of *B. pertussis* have emerged (40). The fitness advantage conferred by loss of this antigen was demonstrated in a mouse model of infection, wherein strains lacking PRN could colonize the lungs at a higher rate and sustain colonization longer than PRN-sufficient strains in aP vaccinated mice (41). Recently, strains deficient in another aP antigen, filamentous hemagglutinin, have also arisen (42). Targeting gene families as a vaccine antigen selection strategy, even when individual antigens are not essential for virulence, increases the potential for retaining protection even if a mutation were to develop. In the case of the Cda family in *C. neoformans*, our data show other members within that family cross-react immunologically and provide cross-protection. An additional attractive feature of targeting the Cda family is strains deficient in Cda1, Cda2, and Cda3 are avirulent (5). In conclusion, our studies lend a strong rationale for inclusion of Cda family proteins in subunit cryptococcal vaccines and provide a proof-of-principle for vaccine antigen selection strategies that target family members, which are highly expressed *in vivo*.

## Data availability statement

The original contributions presented in the study are included in the article/**Supplementary Material**, further inquiries can be directed to the corresponding author.

## Ethics statement

The animal study was reviewed and approved by The University of Massachusetts Chan Medical School Institutional Use and Care of Animals Committee.

## Author contributions

MH, CS, SL, LO, RW, and GO conceived and designed experiments. MH, DL, and ZM performed experiments. MH, CS, and SL analysed the data. MH, CS, and SL wrote the paper. All authors contributed to the manuscript and approved the submitted version.

## Funding

We acknowledge National Institute of Allergy and Infectious Diseases, National Institutes of Health grants AI025780, AI102618, AI125045, and AI072195, and contract 75N93019C00064. MH was partially supported by NIH Training Grant T32 AI095213.

## Acknowledgments

The authors gratefully acknowledge Hiten Madhani and the FGSC for providing the Cpd1, Mep1, and Lhc1 deletion strains. Support for constructing the Madhani Deletion Collection was provided by NIH grant R01AI100272. The authors thank Dennis Christenson (Staten Serum Institut) for helpful advice regarding the use of CAF01, and Ambily Abraham and Florentina Rus for their assistance in preparing the GP vaccines.

## Conflict of interest

The authors declare that the research was conducted in the absence of any commercial or financial relationships that could be construed as a potential conflict of interest.

## Publisher’s note

All claims expressed in this article are solely those of the authors and do not necessarily represent those of their affiliated organizations, or those of the publisher, the editors and the reviewers. Any product that may be evaluated in this article, or claim that may be made by its manufacturer, is not guaranteed or endorsed by the publisher.

## Abbreviations

aP, acellular pertussis; Cpd, carboxypeptidase; CNS, central nervous system; CFU, colony forming units; CSF, cerebrospinal fluid; CM, cryptococcal meningitis; Cda, chitin deacetylase; DPI, days post infection; GP, glucan particle; IFNγ, interferon-gamma; MSA, mouse serum albumin; NCBI, National Center for Biotechnology Information; OVA, ovalbumin; PRN, pertactin; WT, wild-type.
