# Supplementary material for: Cross-reactivity between vaccine antigens from the chitin deacetylase protein family improves survival in a mouse model of cryptococcosis
Source: Front Immunol. 2022 Sep 28;13:1015586. doi: 10.3389/fimmu.2022.1015586 (PMC9554598; doi:10.3389/fimmu.2022.1015586)
Supplement: Supplementary file 1 [file DataSheet_1.docx]

Supplementary Material

**Figure S1:** **Homology of carboxypeptidase family proteins from *C. neoformans* H99**. Alignments were generated by CLUSTAL multiple sequence alignment by MUSCLE (3.8) (22), and they include amino acids 22-548 for Cpd1 (corresponding to the amino acid sequence of the Cpd1 vaccine antigen used in the studies), 22-543 for Cpd2, and 22-541 for Cpd3. Pink highlighting indicates residues are conserved across all three proteins, blue highlights are when two out of the three proteins have a conserved residue, and no highlighting means the three do not have an amino acid in common.

**Figure S2:** **Homology of chitin deacetylase family proteins from *C. neoformans* H99.** Alignments were generated by CLUSTAL multiple sequence alignment by MUSCLE (3.8) (22), and they include amino acids 20-374 for Cda1, 20-328 for Cda2, 19-336 for Cda3, and 20-249 for Fpd1 (corresponding to the amino acid sequences of the vaccine antigens used in these studies). Pink highlighting indicates residues conserved across all four proteins, blue highlights are when three out of four have a conserved residue, grey highlights represent conservation of two out of four residues, and no highlighting means the four do not have an amino acid in common.

**Figure S3: Alignment of Cda2 peptides to the entire Cda family of proteins.** Alignments were generated by CLUSTAL multiple sequence alignment by MUSCLE (3.8) (22), as in **Figure S2**. Pep1 through Pep8 indicate the synthesized Cda2-derived peptides. Pink highlighting indicates residues conserved across all four recombinant proteins (Cda1, Cda2, Cda3, and Fpd1), blue highlights are when three out of four have a conserved residue, grey highlights represent conservation of two out of four residues, and no highlighting means the four do not have an amino acid in common.
